# Efforts of subthalamic nucleus deep brain stimulation on cognitive spectrum: From explicit to implicit changes in the patients with Parkinson's disease for 1 year

**DOI:** 10.1111/cns.13392

**Published:** 2020-05-21

**Authors:** ZhiFei You, Yi‐ Ying Wu, Rong Wu, Zhi‐Xiang Xu, Xi Wu, Xiao‐Ping Wang

**Affiliations:** ^1^ Department of Neurology Shanghai General Hospital of Nanjing Medical University Shanghai China; ^2^ Department of Neurology Shanghai TongRen Hospital Shanghai Jiao‐Tong University School of Medicine Shanghai China; ^3^ Department of Neurosurgery Changhai Hospital The Second Military Medical University Shanghai China

**Keywords:** cognitive function, deep brain stimulation, explicit memory, implicit memory, neuromodulatory, Parkinson's disease, subthalamic nucleus

## Abstract

**Objective:**

To evaluate the cognitive function of Chinese patients with Parkinson's disease PD postsubthalamic nucleus deep brain stimulation (STN‐DBS).

**Methods:**

Cognitive function was assessed by neuropsychological methods in PD patients. Twenty matched healthy persons served as normal controls. *t* test, analysis of variance, and chi‐square analysis were used to compare the difference among the groups. Reliable change index was utilized to analyze the changes in cognition from the individual level.

**Results:**

(a) Improvement in motor function was significantly better after STN‐DBS (*P* < .01). (b) Notably, the increase error rates of implicit SRTT (serial reaction time task) was significantly higher after STN‐DBS as compared with the conservative therapy group (*P* = .03). (c) The decline of verbal fluency (explicit) was also significantly higher after STN‐DBS than that in the medication therapy group (*P* = .03). (d) In the explicit clock‐drawing test, scores had significantly improved after STN‐DBS (*P* = .04).

**Conclusions:**

STN‐DBS as a neuromodulatory tool in the Chinese PD population not only improves motor symptoms but also cognitive function to a certain extent, such as the decline of executive function and verbal fluency. The explicit cognitive decline was significantly quicker than that in patients on medication therapy. The improvement of visiospatial function was also noted. Implicit memory impairment during the 1‐year follow‐up period was not observed.

## 1 INTRODUCTION

Parkinson's disease (PD) refers to the disruption of the motor loop between the basal ganglia and motor cortex induced by the loss of dopaminergic neurons in the substantia nigra, resulting in resting tremor, muscle tension increase, slow motion, and postural disturbance.^1^ PD has become a common neurodegenerative disease in middle‐aged and elderly people. Besides motor symptoms, PD patients also show nonmotor symptoms, such as cognitive impairment, autonomic dysregulation, and sleep disorders, which seriously affect the quality of their life.^2^ Currently, deep brain stimulation (DBS) is gradually being used on a large scale, owing to the significant success it has shown in improving motor symptoms, quality of life, and reducing daily drug dependency.^3^, ^4^

Effects of STN‐DBS on PD patient's cognitive function are still not understood. It is believed that STN‐DBS significantly alleviates motor symptoms through cortex‐basal ganglia‐thalamus‐cortex loop modulation.^5^, ^6^ However, dose STN‐DBS have similar effect on the cognitive nerve loop? As an increasing number of PD patients choose DBS, there has noticeably an arising postoperative complications particularly relating to cognitive function. A randomized, double‐blind clinical trial confirmed improvement of STN/globus pallidus internus (GPi) DBS on motor symptoms, but found decrease in memory, verbal fluency, and visuospatial function in PD patients after DBS.^7^ However, Halpern et al^8^ believed that working memory and psychomotor speed were improved after surgery, and that only verbal fluency and executive function decreased. Williams et al^9^ found that the incidence of dementia was significantly higher in the STN‐DBS group (32%) than that in the optimal medication therapy group (16%) after 2 years of follow‐up. Aybek et al^10^ reported that postoperative dementia is a natural progression of PD.

Memory is one of the key components of cognition. Previous studies have shown that STN‐DBS could interfere working memory.^11^, ^12^ It has been reported, instances of some patients that could not recognize facial stimuli ^13^or discern vocal emotion.^14^ We supposed STN‐DBS might impact patients' implicit memory and cause some slight cognitive change. This study attempts to explore the effects of STN‐DBS on cognitive function and its potential mechanism by means of a specific neuropsychological tool.

## 2 SUBJECTS AND METHODS

The ethics committee of Shanghai Jiao Tong University School of Medicine approved the study (for project number 81071065#). The authors received written informed consent from every participant.

### 2.1 Patients and groups

Forty‐three PD patients were selected from the Shanghai JiaoTong University School of Medicine in China from August 2013 to August 2015. All subjects were in accordance with the diagnostic criteria for idiopathic Parkinson's disease of United Kingdom Parkinson's Disease Society Brain Bank.^15^ None of the patients met the following conditions: (a) invalid dopaminergic therapy; (b) severe psychiatric symptoms, such as schizophrenia symptoms, apathy, suicidal tendencies, and severe depression; (c) history of stroke, brain injury, and intracranial infection; (d) severe heart, liver, lung, kidney, and blood system, endocrine system diseases; (e) history of drug abuse. Three cases were dropped out due to good motor symptoms control and patients delayed follow‐up time point more than 3 months. The drop‐out rate was less than 10%. Twenty PD patients received bilateral STN‐DBS in the Department of Neurosurgery as the STN‐DBS group (DBS group). And twenty PD patients without surgery were matched with STN‐DBS group, received the optimal medication therapy as the medication therapy group (MED group).Twenty (10 females) matched healthy old people, composed the control group, with average age 61.1 ± 5.12 years, education time 8.30 ± 4.95 year). No difference about gender, age, and education time between groups.

**Figure 1:**
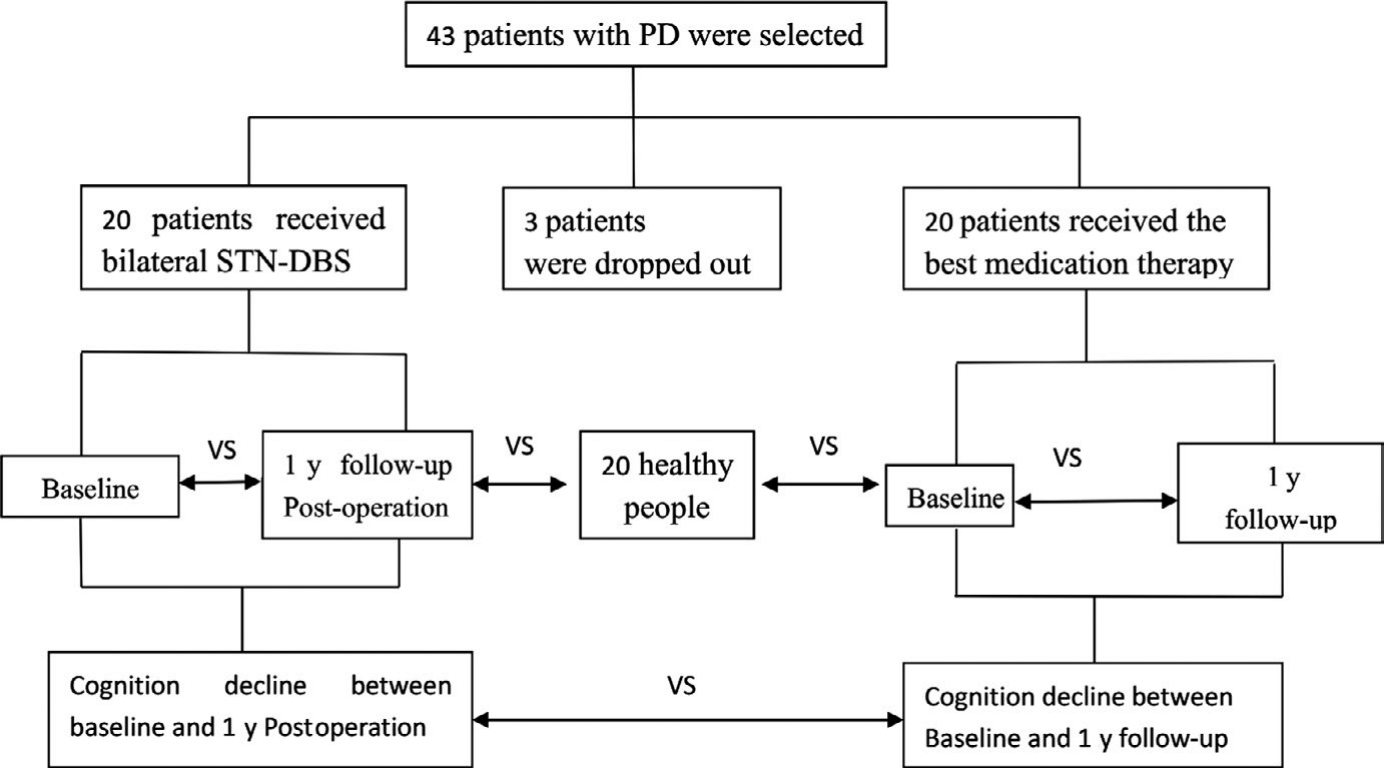
Flowcharts of the study designed

All the patients were evaluated for motor symptoms and cognition impairment at the beginning of the study and after 1‐year follow‐up. As a control, 20 healthy candidates were also evaluated for cognition impairment. This control group was then compared with DBS and MED group at baseline. The declination of cognition between DBS group and MED group was compared, read the flowcharts in Figure 1.

### 2.2 Clinical evaluation

#### 2.2.1 Assessment of motor symptoms

Unified Parkinson's Disease Rating Scale^16^ and Modified Hoehn&Yahr stage (H‐Y stage)^17^ were assessed by two certificated neurologists (Dr Wu Xi and Dr Qiu Yiqing) to evaluated patients' motor symptoms at “On time” and “Off time” (patient was not taken medication for 12 hours).

#### 2.2.2 Assessment of cognition

Tests were administered by the neuropsychologist (Dr Wang Xiaoping), included Mini Mental State Examination (MMSE),^18^ Wechsler Memory Scale,^19^ Verbal fluency, Clock‐drawing, Degraded picture naming, and serial reaction time task(SRTT).^20^ The assessment of cognition was launched at “On time” on the day after motor symptoms assessment. The SRTT software version can count response time (T_avg_), error rates, and minimum reaction time (T_min_). Attention is quantified by minimum reaction time (T_min_).

### 2.3 STN‐DBS operation and confirm the leads localization

#### 2.3.1 Operative procedure

All surgeries performed with preoperative cranial 3.0T MRI and standardized stereotactic procedures. The stereotactic apparatus was Leksell G frame (Elekta). The coordinates calculated and trajectory designed with Medtronic S7 Neuro Navigation System (Medtronic Navigation) to target the STN. Bilaterally Medtronic DBS electrodes (Model 3389 electrode; Medtronic) implanted under local anesthesia and IPG (Activa PC; Medtronic) implanted under general anesthesia. All the operations achieved in 1 day. Postsurgery stimulation parameters were set 4 weeks postoperation and gradually achieved the optimal stimulation parameters in 3 months.

#### 2.3.2 Leads localization

The Medtronic S7 Neuro Navigation System (Medtronic Navigation) was used to merge preoperative 3.0T‐MRI images with postoperative CT images. The CT scan images were calibrated at 1 mm thickness and without pneumocranium. The Lead‐DBS software^21^ was used to show the electrodes and active contacts the same as our previous work done.^22^

### 2.4 Statistical analysis

Comparison of the overall level—Data were analyzed with SPSS 19.0 software. Measurement data were expressed as mean ± SD or min‐max. A value of *α* = .05 was considered statistically significant difference. Self‐control group was evaluated with paired *t* tests. Group comparison evaluated with student *t* test. Data that not obey normal distribution were analyzed with Mann‐Whitney *U* rank sum test. Count data were compared by using chi‐square test.

Comparison of the individual level—Some individual cognitive differences may be masked after averaging. Several reliable change indices (RCIs) exist to evaluate statistically significant individual change with repeated neuropsychological assessment. We present reliable change indices (RCIs) to compare statistically reliable cognitive changes between the groups for each neuropsychological measure over time.^23^ According to the reliable change indices (RCIs), RCI = (X2−X1)/SE_diff_, where X1 is the patient's baseline score, X2 is the patient's 1‐year score, and S_diff_ is the standard error of the difference between the test scores. RCI > 1.96 represents remarkable changes before and after test.

## 3 RESULTS

### 3.1 Two patients' groups

STN‐DBS group and MED group were both including 20 patients (10 female and 10 male). Two groups had no significant difference at gender, age, disease duration, H –Y grade, UPDRS sore, and LED at baseline (see Table 1).

**Table 1:** Clinical data for patients

Baseline data	STN‐DBS Group	MED Group	*P* value
Number of patients	20	20	
Gender	M10/F10	M10/F10	1.00
Age	59 ± 4.23	58.35 ± 6.77	.718
Disease duration	9.55 ± 2.35	8.65 ± 2.3	.229
H‐Y grade(med off)	2.5(2)3(13)4(3)5(2)	2.5(3)3(14)4(2)5(1)	.401
UPDRS‐Ⅰ	4.15 ± 1.31	4.85 ± 2.28	.241
UPDRS‐Ⅱ(med off)	19.2 ± 4.65	18.55 ± 5.73	.696
UPDRS‐Ⅲ(medon)	22.3 ± 10.7	20.6 ± 7.32	.670
UPDRS‐Ⅲ (medoff)	48.5 ± 14.4	46.70 ± 11.1	.560
UPDRS‐Ⅳ	5.90 ± 2.36	5.85 ± 2.13	.944
LED	785 ± 236	773 ± 352	.906
1‐y follow‐up			
UPDRS‐Ⅲ (medon)		20.9 ± 7.13	
UPDRS‐Ⅲ (medoff IPG on)	24.4 ± 8.71		
UPDRS‐Ⅲ (medoff IPG off)	45.8 ± 10.4	47.3 ± 11.1	
LED	407 ± 197	832 ± 260	

Note

Gender compare used Fisher's exact probability. Medoff: Off time that stopped anti‐parkinsonism medication at least 12 h; Medon: On time with anti‐parkinsonism medication.

Abbreviation: LED, Levodopa equivalent dose; MED, medication therapy; STN‐DBS, subthalamic nucleus deep brain stimulation.

### 3.2 Deep brain stimulation

**Figure 2:**
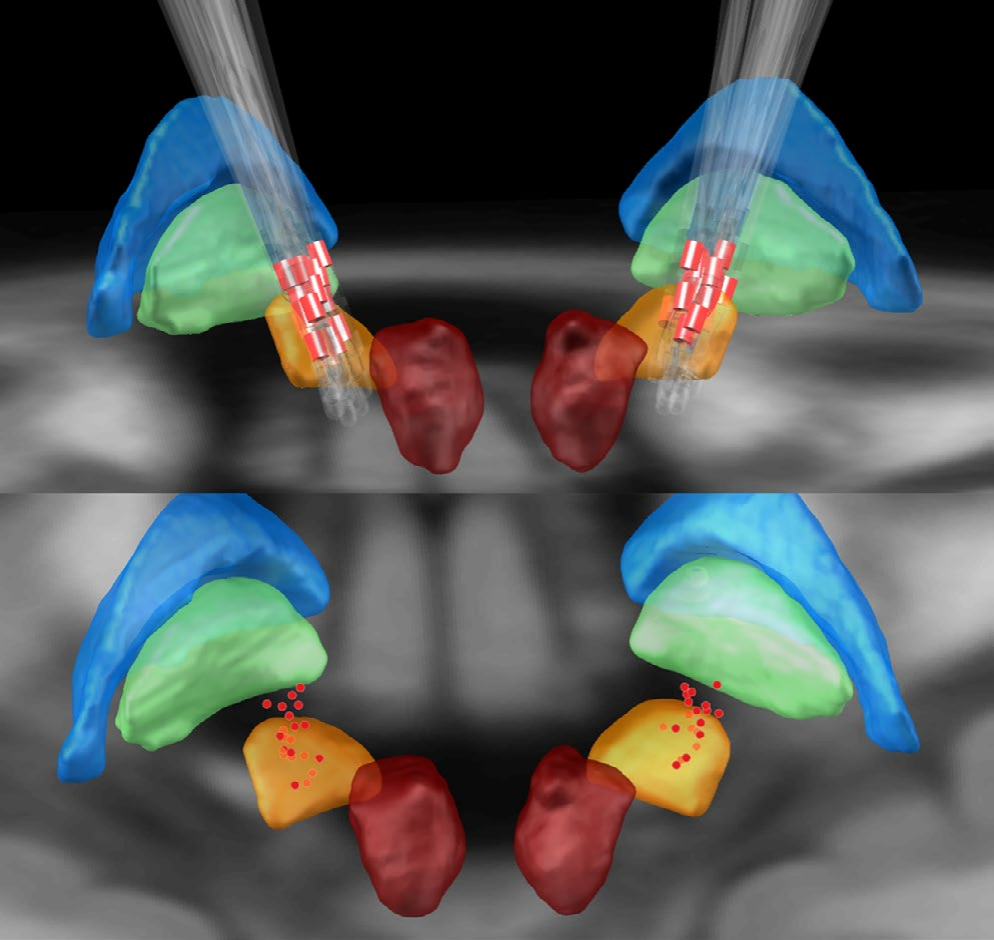
Location of leads and active contacts. The figure showed red nucleus (dark red), subthalamic nucleus (yellow), internal globuspallidus (green), and external globuspallidus (glue). The active contacts were red, and their centers were marked as red spots

All 20 STN‐DBS patients had no surgical and hardware‐related complications after 1‐year follow‐up. Two of the patients gradually abandon anti‐parkinsonism medication after the optimal stimulation. The location of electrodes and active contacts were shown in Figure 2. Nineteen patients and 39 electrode used monopolar stimulation, but one patients' left electrode used interleaving mode for speech stuttering. The average stimulation parameters were amplitude of 2.35 ± 0.20 volts, frequency of 130 ± 28.5 hertz, and pulse width of 74.6 ± 17.5 microseconds.

### 3.3 Assessment of motor symptoms

No significant difference in UPDRS scores was detected between the DBS group and MED group at baseline. As displayed in Table 1, motor function were significantly improved 49.7% in the DBS group postoperation (MED off IPG on) compared with that preoperation (MED off) (*P* < .001), postoperative required drug dose significantly decreased 48.2% (*P* < .001). Two patients totally stop taking medication at the optimal DBS stimulation.

### 3.4 Assessment of cognitive function

#### 3.4.1 Memory

**Figure 3:**
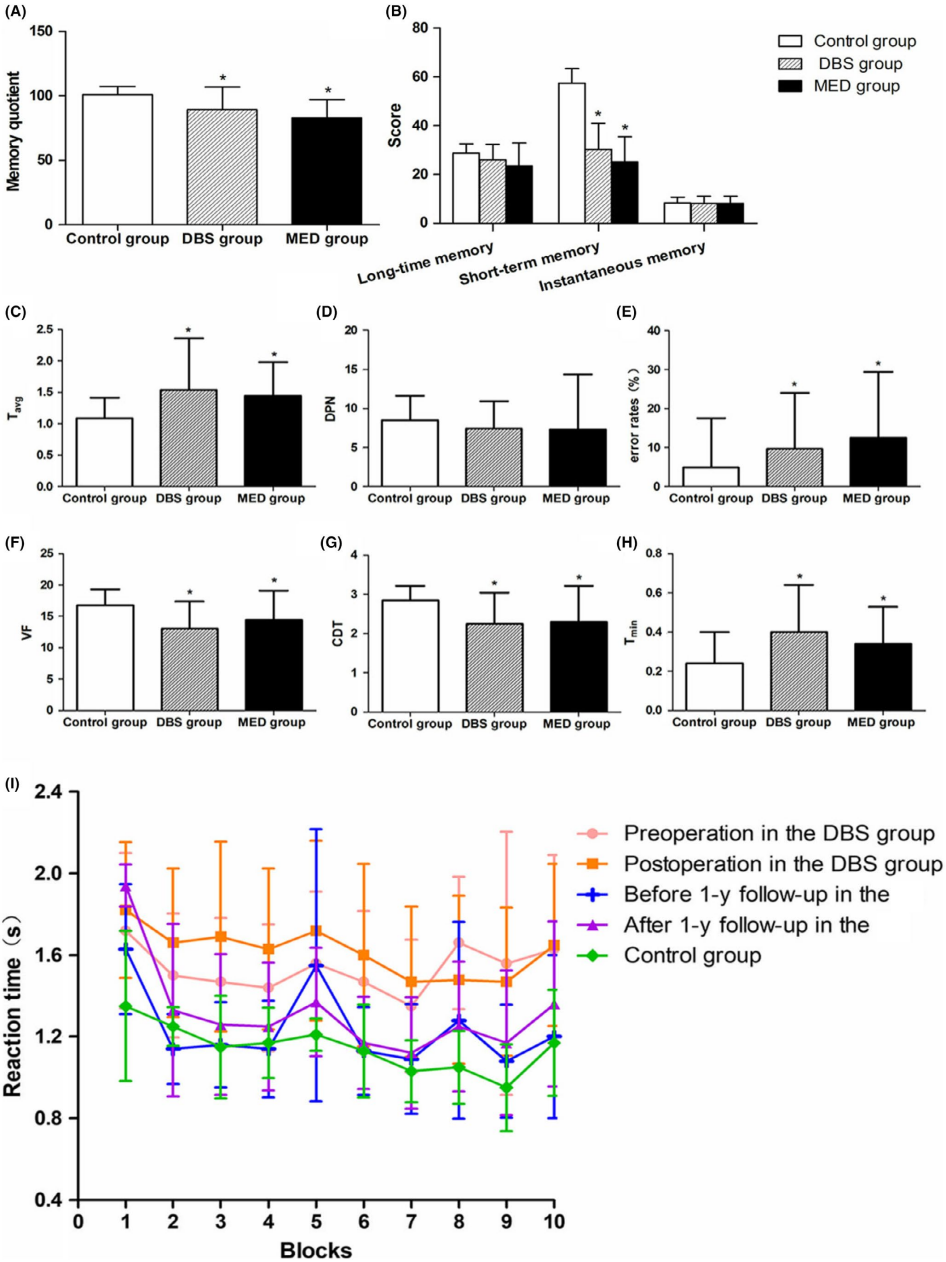
Comparison of memory and cognitive index in each group at baseline and line graph of response time in each group. Note: *P < .05, vs control group

Memory quotient measured by Wechsler Memory Scale reflects explicit memory. Student‐Newman‐Keuls pairwise comparison test results showed that memory quotient was significantly lower in the DBS and MED groups than in the control group (F = 8.62, *P* < .01; Figure 3A). There was no significant difference in long‐term memory and instantaneous memory obtained by the superposition of the scale of the same type of subtest among DBS, MED and control groups (F = 2.13, *P* > .05；F = 1.54; *P* > .05). Short‐term memory was significantly decreased in the DBS and MED groups compared with the control group (F = 4.56, *P* < .05; Figure 3B).There was no significant difference in memory quotient and each subtest before and after surgery in the DBS group (*P* > .05). There was no significant difference in memory quotient and each subtest before and after 1‐year follow‐up in the MED group (*P* > .05). And there was no significant change in the range of memory quotient of the two groups. Changes in memory quotient were considered as an observed value in the DBS and MED groups before and after 1‐year follow‐up. No significant difference in the change was detected between the two groups (F = 0.07, *P* = .800; Table 2).

**Table 2:** Comparison of cognitive spectrum in each group

Item	Control group	DBS group (Preoperatively)	DBS group (Postoperatively)	MED group (1‐y follow‐up)	MED group (1‐y follow‐up)
Memory quotient (min‐max）	101 ± 6.45	89.4 ± 17.41* (55‐126）	89.2 ± 17.2* (60‐128)	82.9 ± 14.0* (51‐110)	81.6 ± 17.1* (52‐108)
Change		−0.10 ± 9.21		−1.24 ± 10.7	
T_avg_ (second)	1.09 ± 0.32	1.54 ± 0.82* (0.73‐4.42）	1.62 ± 0.80 (0.73‐3.39）	1.44 ± 0.54* (0.71‐4.02)	1.50 ± 0.65 (0.72‐3.04)
Change		0.025 ± 0.53		0.05 ± 0.56	
DPN	8.50 ± 3.09	7.40 ± 3.56 (4‐22)	7.60 ± 3.09 (6‐25)	7.30 ± 3.00 (3‐19)	7.05 ± 3.20 (2‐20)
Change		0.2 ± 2.00		0.2 ± 2.00	
Error rate (%)	4.84 ± 12.7	9.71 ± 14.3* (1.7‐66.1)	13.4 ± 10.7* (1.2‐40.9)	12.6 ± 16.9* (1.1‐69.1)	9.50 ± 12.0 (0.8‐58.8)
Change		3.72 ± 12.6		−3.05 ± 6.56^▲^	
Verbal fluency	16.8 ± 2.54	13.1 ± 4.31* (6‐23)	11.7 ± 2.86* ^,^ ^#^ (10‐24)	14.4 ± 4.69* (6.5‐25)	14.3 ± 6.21* (5‐24)
Change		−1.35 ± 0.85		−0.85 ± 3.58^▲^	
Clock‐drawing test	2.85 ± 0.36	2.25 ± 0.79* (0‐3)	2.70 ± 0.57^#^ (0‐3)	2.30 ± 0.92* (0‐3)	2.05 ± 0.83* (0‐3)
Change		0.45 ± 0.89		−0.25 ± 0.85^▲^	
T_min_ (second)	0.24 ± 0.16	0.40 ± 0.24* (0.02‐0.78)	0.28 ± 0.21^#^ (0.02‐0.75)	0.34 ± 0.19* (0.04‐0.72)	0.28 ± 0.20 (0.02‐0.70)
Change		−0.12 ± 0.22		−0.08 ± 021	
Mini Mental State Examination	27.6 ± 1.79	26.9 ± 2.07	26.8 ± 2.57	25.8 ± 4.19	26.1 ± 3.12

Note

Change = postoperation—preoperation or after 1‐y follow‐up—before 1‐y follow‐up. In the clock‐drawing test, Mann‐Whitney *U* rank sum test was used to compare the difference among DBS, MED, and control groups. Paired rank sum test was utilized to compare the difference before and after surgery or 1‐y follow‐up.

* *P* < .01, vs control group;

^#^ *P* < .05, vs preoperation;

^▲^ *P* < .05, vs DBS group using MANOVA.

The SRTT was a task of procedural learning. Analysis of variance showed significant differences in T_avg_ among DBS group, MED group and control group (F = 4.90, *P* < .05). Student‐Newman‐Keuls pairwise comparison test results showed that T_avg_ was significantly higher in the DBS and MED groups than the control group. No significant difference in T_avg_ was detectable between the MED and DBS groups at baseline (Figure 3C). Analysis of variance results did not show significant differences in the number of degraded picture naming among the DBS and MED groups at baseline and control group (F = −1.19, *P* = .23; Figure 3D). There was no significant difference in T_avg_ among different blocks in the DBS and MED groups (preoperatively F = 0.28, *P* = .98; postoperatively F = 0.37, *P* = .95; 1 year before medication therapy F = 1.18, *P* = .32; 1 year after medication therapy F = 1.86, *P* = .06). In the control group, T_avg_ displayed reduced across the blocks of repeated sequence trials, until to block 5 the rule disappeared, subsequent T_avg_ increased gradually. After the rule restored again, T_avg_ reduced again. It conformed to the learning rule of implicit memory inhuman. Except patients after DBS, the remaining PD patients presented significantly prolonged T_avg_ from the block 8. This indicates that after repeated operation, PD patients entered the fatigue period. There is no such phenomenon in the test after DBS, and a curved shape is close to the control. Our results demonstrated that T_avg_ had an increasing trend after DBS, but the difference was not statistically significant (t=−0.81, *P* = .43). T_avg_ showed an increased trend in the MED group compared with that after 1‐year follow‐up, also no significantly difference (t=−0.31, *P* = .76). There were no significant differences in the number of degraded picture naming between the two groups (t=−0.450, *P* = .66; t = 0.324, *P* = .71). T_avg_ increments were (0.025 ± 0.53) seconds and (0.05 ± 0.56) seconds after 1‐year following up in the DBS and MED groups, respectively. Considering levodopa equivalent dose altered before and after 1‐year follow‐up, multivariate analysis of variance indicated that there was no significant difference in the increased trend of T_avg_ in both groups (F = 0.001, *P* = .98; Table 2).

### 3.5 Executive function

Error rates in serial reaction time task in the control group, before and after DBS in the DBS group, before and after 1‐year follow‐up in the MED group were (4.84 ± 12.7)%,(9.71 ± 14.3)%, (13.4 ± 10.7)%, (12.6 ± 16.9)%, and (9.50 ± 12.0)%, respectively, there was not any significant difference in error rate detected in DBS and MED groups, compared with it at baseline(t=−1.31, *P* = .21 in the DBS group; t = 1.97, *P* = .07 in the MED group) after a 1‐year follow‐up period. The increased trend of error rate was significantly higher in the DBS group after surgery compared with the MED group (F = 6.16, *P* = .03; Table 2).

### 3.6 Speech function

There was a significant difference in verbal fluency among the control group, DBS group (preoperative) and MED group (before 1‐year follow‐up) (*P* < .01). This could have been as a declined language function in PD patients (Figure 3F). Verbal fluency decreased after DBS (t = 2.48, *P* < .05). No significant difference in verbal fluency was determined between 1‐year follow‐up and baseline data in the MED group (t=−1.06, *P* = .302). The decreased trend was significantly higher in the DBS group than in the MED group (F = 6.27, *P* = .03; Table 2).

### 3.7 Visuospatial ability

Clock‐drawing test showed a significantly lower test score in the DBS and MED groups at baseline compared to the control group (DBS group: z = −2.76, *P* < .01; MED group: z = −2.55, *P* < .01; Figure 3G). The score was significantly increased after surgery in the DBS group (z = −2.07, *P* < .05). Moreover, no significant difference was detected between DBS group postoperatively and control group, suggesting that postoperative clock‐drawing test results were close to normal levels. Clock‐drawing test score was decreased after 1‐year follow‐up in the MED group, but the difference was not statistically significant (z = −1.31, *P* = .19). Variations were compared between the two groups, multivariate analysis of variance of levodopa equivalent dose variation demonstrated significant difference (F = 5.71, *P* = .04; Table 2).

### 3.8 Attention

T_min_ was significantly increased in the DBS and MED groups at baseline compared with the control group (t = 2.41, *P* < .05; Figure 3H). T_min_ had a significant decrease after BDS (t = 2.83, *P* < .05). T_min_ also exhibited a decreased trend in the MED group after 1‐year follow‐up, but no significant difference (t = 1.18, *P* = .26). No significant difference in variations was found between the two groups (F = 2.83, *P* = .12) (Table 2).

### 3.9 Comparison of reliable change index from the individual level

Each test values were calculated in the DBS and MED groups based on reliable change index. The declined, remained stable, and improved percentages of each test are listed in Table 3. Significant differences on verbal fluency and error rate were seen between the DBS and MED groups (x^2^ = 7.03, *P* = .03; x^2^ = 6.72, *P* = .04).

**Table 3:** Declined, remained stable, and improved percentages in each test in the DBS and MED groups

	DBS group			MED group		
	Declined (%)	Remained stable (%)	Improved (%)	Declined (%)	Remained stable (%)	Improved (%)
Clock‐drawing test	0	85	15	10	90	0
Verbal fluency*	20	80	0	0	85	15
Degraded picture naming	0	95	5	5	85	10
Error rate*	5	75	20	30	70	0
T_avg_ (second)	10	85	5	0	100	0
T_min_ (second)	20	80	0	12	88	0
Memory quotient	10	85	5	12	76	12

Abbreviations: DBS, deep brain stimulation; MED, medication therapy.

* *P* < .05 in chi‐square test.

## 4 DISCUSSION

Several and nonexclusive mechanisms have been proposed to underlie the beneficial effects of DBS, such as electrical and neurochemical effects of stimulation, modulation of oscillatory activity, synaptic plasticity, and, potentially, neuroprotection and neurogenesis.^24^ It is generally believed that cognitive function is closely associated with the prefrontal lobe‐basal ganglia‐thalamic circuit.^25^ Any affected link on the loop will cause the change of cognitive function, which may be the basis of STN‐DBS affecting the cognitive function of PD patients. The patients with PD are facing the damage of cognition, mainly involving memory, executive function, attention, and verbal fluency. To investigate the cognitive changes after STN‐DBS is the natural cognitive outcome of PD or the impact of DBS and the potential pathogenesis, this study explored the change of cognitive spectrum of PD patients after operation by using a variety of neuropsychological assessment scales.

Our results demonstrated that explicit memory was really impaired in PD patients, mainly in short‐term memory impairment. However, DBS did not noticeably affect explicit memory compared with patients that received the simple medication therapy. And Rothlind et al^26^ agreed with us on the change of explicit memory. With the in‐depth study of memory, a theory of implicit memory is presented which describes it as a form of general plasticity within processing networks that adaptively improve function via experience.^27^ The line graph of normal controls in serial reaction time task test showed the priming effect of implicit memory on later learning. Compared with the health, implicit memory was impaired in PD patients, but STN‐DBS did not evidently deteriorate implicit memory. Different types of memory have different neural structures and loop. The medial temporal lobe and the hippocampus are involved in explicit memory, while basal ganglia is involved in implicit memory.^28^ Explicit and implicit memory systems act the memory function through different cognitive mechanisms, but they influence each other.^29^ The basal ganglia are likely to be the linked between the two memory systems.^30^ However, the effects of basal ganglia on the memory system and its relationship with explicit and implicit memories deserve further investigations. It is emphasized that the healthy control group also was scored by the implicit and explicit cognition tests, so to observe and adjust the age‐dependent tendency of natural cognitive outcomes for a year.

Verbal fluency decline is an important cognitive impairment in PD patients. Regarding the changes in verbal fluency after DBS, the views of scholars are mainly divided into two factions: (a) The decrease in verbal fluency after DBS is associated with cognitive decline in PD.^9^, ^31^ (b) DBS induced a decrease in verbal fluency.^32^, ^33^ Our results confirmed that STN‐DBS definitely has a bad effect on verbal fluency. Its internal mechanism may include micro‐damage after surgery or current stimulation. It was demonstrated that PD patients do experience executive dysfunction and STN‐DBS worsened the decline of executive function, which is not the natural cognitive outcome in PD. In addition, we found that executive function obviously decreased in 20% patients after surgery. Smeding et al^30^ and Williams et al^9^ assessed executive function with other neuropsychological measures and got the same conclusions. Nevertheless, effect of STN‐DBS on executive function of PD patients still needs more data to support. Visuospatial dysfunction is also considered common cognitive impairment in PD patients (especially the middle stage and the advanced). Results exhibited that STN‐DBS improved visuospatial function to a certain degree and 15% patients had improved after DBS. It is believed that visuospatial function is related to the visual cortex currently^31^, ^32^, ^33^. Whether the improvement of STN‐DBS on visuospatial function is induced by current diffusion to stimulate visual cortical pathway or change of dopamine concentration after surgery, or other mechanisms, deserves further investigations. Another parameter of cognitive impairment in PD patients was attention. STN‐DBS may improve attention, but cannot completely eliminate the possibility of medication therapy.

In conclusion, STN‐DBS obviously improved motor symptoms, and simultaneously altered cognitive function, such as the decreased executive function and verbal fluency. The decline is more obvious than the natural cognitive decline in patients with PD. Visuospatial function was improved, and attention was possibly improved.

Explicit memory and overall cognitive function did not noticeably alter. Implicit memory was retained, and STN‐DBS did not affect implicit memory in a short period. Long‐term effects need to be made clear by longer follow‐up and more in‐depth study.

## CONFLICT OF INTEREST

The authors declare that the research was conducted in the absence of any commercial or financial relationships that could be construed as a potential conflict of interest.

## ETHICAL APPROVAL

This study was performed in accordance with the guidelines of the Declaration of Helsinki (1964) and was approved by the medical ethics committee JiaoTong University School of Medicine. All subjects provided written informed consent prior to their inclusion in the study.
